# Case Report: Incarcerated femoral hernia of the appendix with incidental discovery of goblet cell carcinoma

**DOI:** 10.3389/fmed.2026.1831458

**Published:** 2026-06-03

**Authors:** Lifei Zhang, Yunna Ma, Yanan Fan

**Affiliations:** 1 Department of Gastrointestinal Surgery, Hebei General Hospital, Shijiazhuang, Hebei, China; 2 Department of Cardiovascular Medicine, Hebei General Hospital, Shijiazhuang, Hebei, China

**Keywords:** appendix, De Garengeot hernia, femoral hernia, goblet cell carcinoma (GCC), laparoscopic appendectomy (LA), transabdominal preperitoneal (TAPP)

## Abstract

**Background:**

Femoral hernias are relatively uncommon in clinical practice but are of considerable clinical significance because they are associated with a higher risk of strangulation happening. Even rarer is the discovery of the appendix within the hernia sac, a condition known as De Garengeot hernia, and within this already highly unlikely scenario, having a tumor within the hernia is exceedingly rare. Among such cases, Goblet cell carcinoma (GCC) represents an incredibly rare occurrence.

**Case summary:**

We present the case of an 83-year-old man who had undergone transabdominal preperitoneal (TAPP) repair for an incarcerated femoral hernia. During the operation, the incarcerated part was found to be the tip of the appendix, which made it necessary to perform a laparoscopic appendectomy (LA). Subsequent histopathological examination revealed GCC invading the muscularis propria, and given the patient’s advanced age, significant comorbidities, and the family’s stated preference, a right hemicolectomy was not performed. At the 1-year follow-up, computed tomography (CT) and tumor markers indicated no sign of recurrence.

**Conclusion:**

For older individuals with co-existing health conditions and those at the early stage of GCC, a cautious management approach backed by active monitoring might be a sensible option following a thorough evaluation of risks and benefits, and this instance provides a realistic view on dealing with such rare and medically complex scenarios.

## Background

Inguinal hernias constitute one of the most common diagnoses in general surgical practice (1), whereas femoral hernias account for only 2–4% of all inguinal hernias and are associated with a significantly heightened risk of incarceration and strangulation (20–50%), which renders them a frequent surgical emergency (2, 3), and De Garengeot hernia is even more uncommon, as it is characterized by the appendix being present within the hernial sac and occurs in fewer than 5% of femoral hernia cases (4). Moreover, encountering a tumor within such a hernia is an extremely rare event, particularly GCC—a pathological entity whose biological characteristics lie somewhere between those of neuroendocrine tumors and typical adenocarcinoma and that is seldom documented in this context. The ideal management of GCC continues to be a matter of debate due to its combined nature and diverse prognosis (5).

In this study, we present the case of an 83-year-old man who presented with an incarcerated femoral hernia and was incidentally diagnosed with GCC as well. We review the challenges in diagnosis and treatment posed by this rare combination and outline our individualized, surveillance-centered treatment plan in view of the present evidence.

## Case presentation

An 83-year-old man presented to the emergency department with complaints of sudden onset of right lower abdominal pain lasting 18 h after a forceful sneeze, and he also reported a painful, swollen, and non-reducible lump in his right groin at the same time.

**Figure 1:**
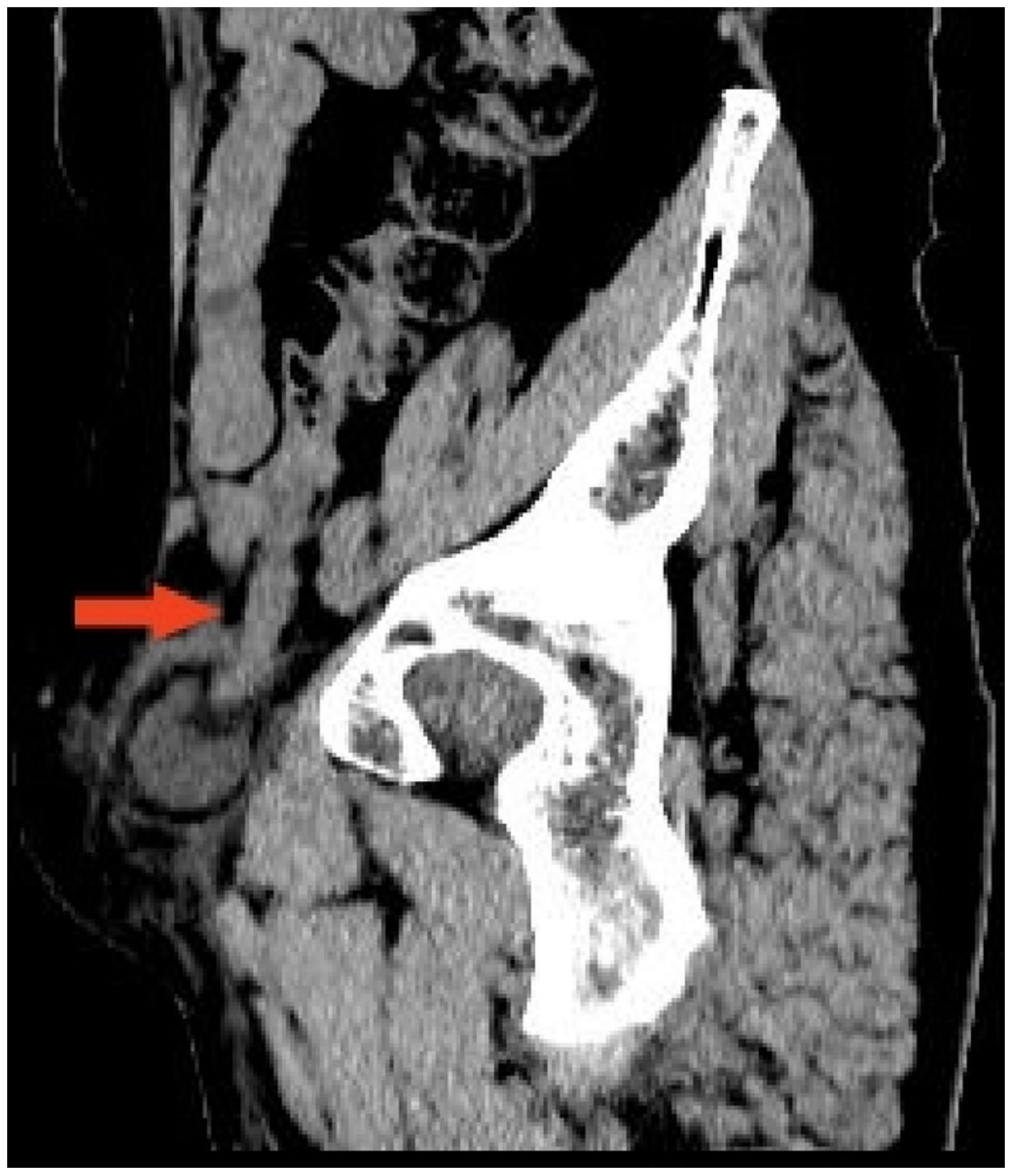
Abdominal CT coronal image of the appendix bursting into the right groin.

**Figure 2:**
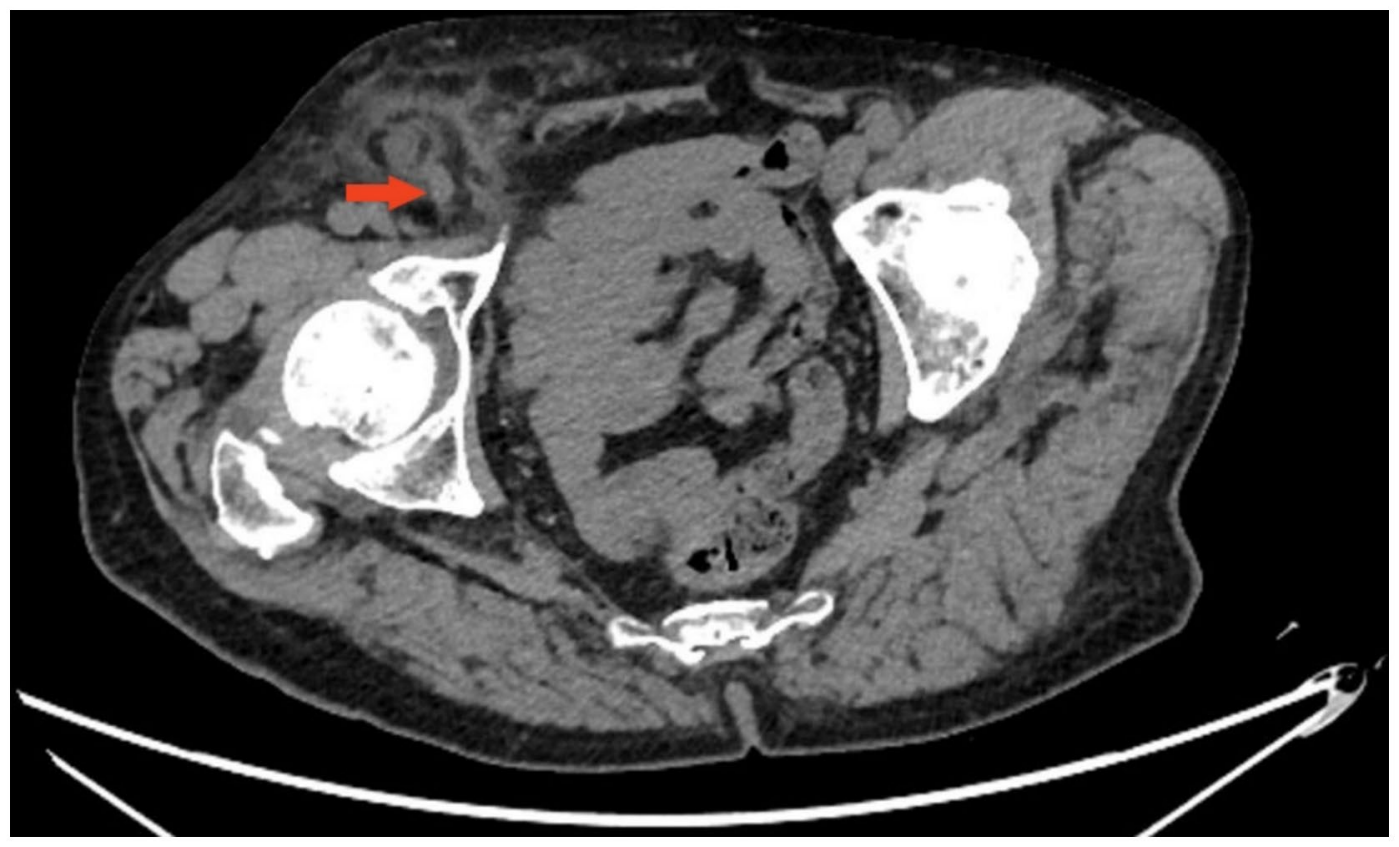
Abdominal CT axial scans reveal an appendix within a right inguinal (Amyand’s) hernia.

During the physical examination, a firm and sore mass measuring 4 cm was palpated in the right femoral area. The skin covering it appeared normal, with no indications of redness, swelling, or sores, and there was no noticeable enlargement of the external inguinal ring. CT scan revealed a right femoral hernia containing indistinct fat layers and the tip of a slightly enlarged appendix. Notably, no abnormal lymph nodes around the appendix were found (Figures 1, 2).

**Figure 3:**
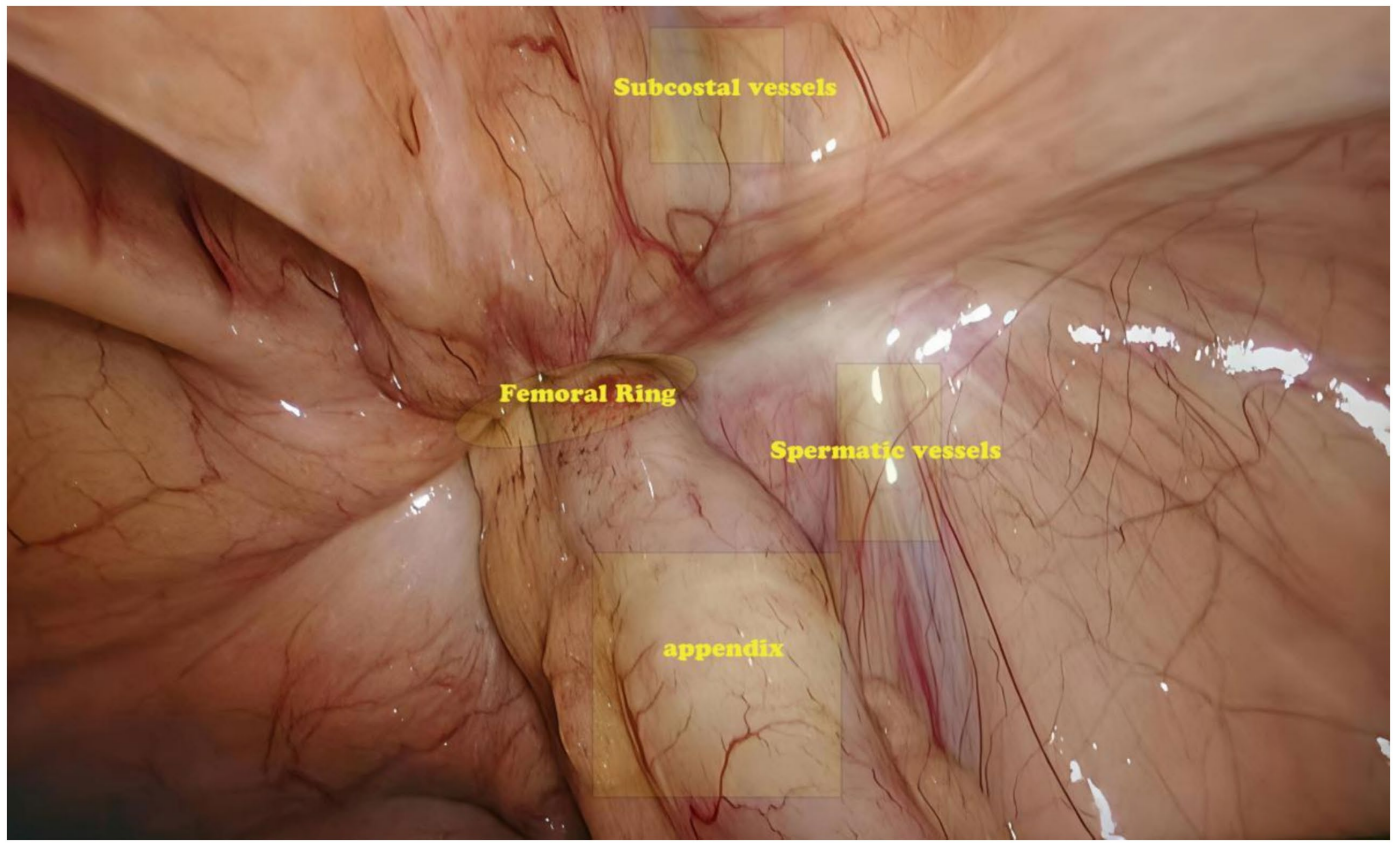
Laparoscopic examination revealed that the tip of the appendix had herniated from the right femoral ring.

**Figure 4:**
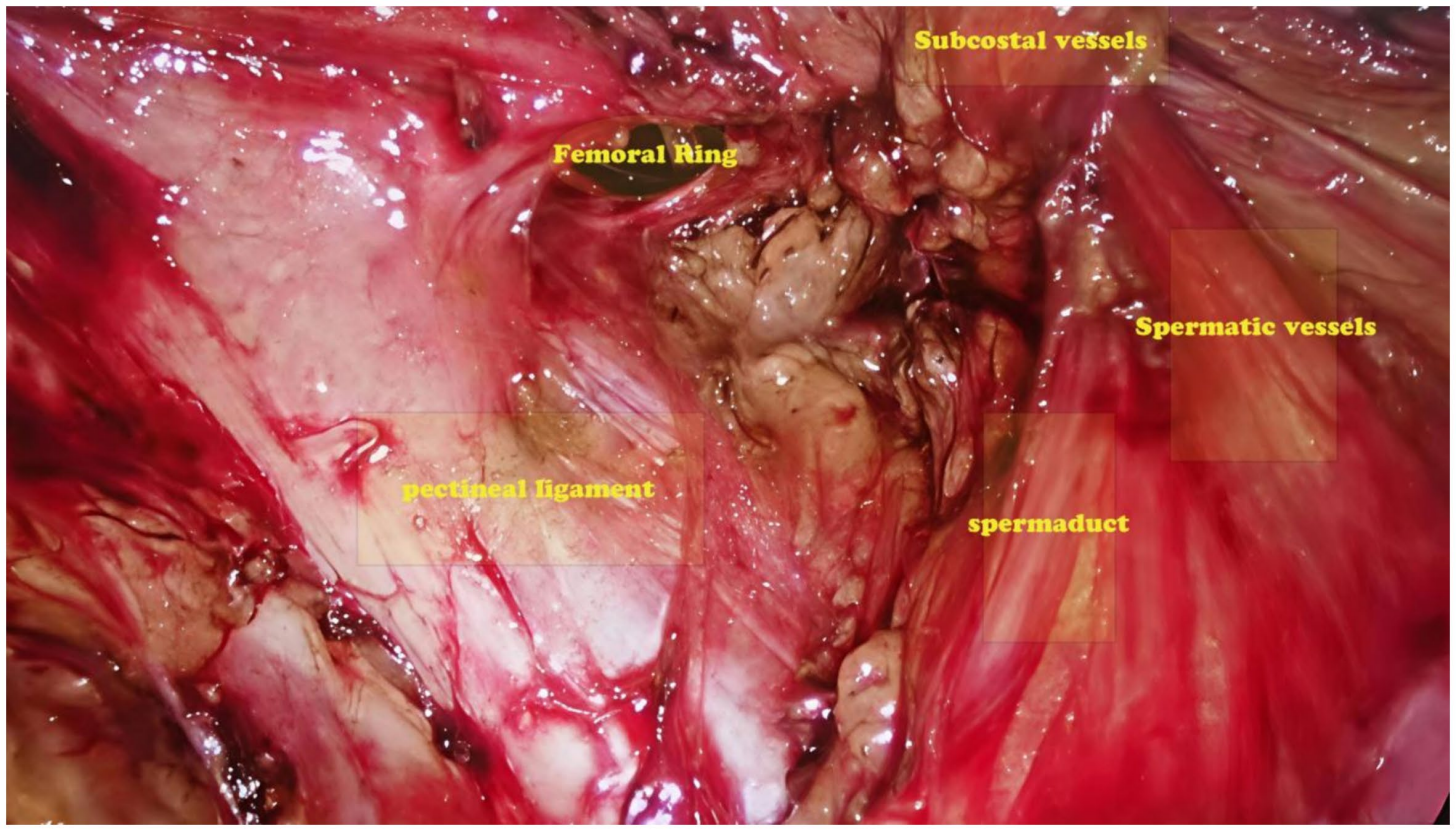
Anterior peritoneal space was dissociated.

Emergency laparoscopy confirmed the presence of a femoral hernia, with incarceration of the tip of the appendix (Figure 3). The herniated portion appeared edematous; however, there was no sign of necrosis or perforation, and approximately 20 mL of slightly cloudy, bloody fluid was observed within the hernia sac. Subsequently, a TAPP hernia repair was performed (Figure 4), followed by a LA.

**Figure 5:**
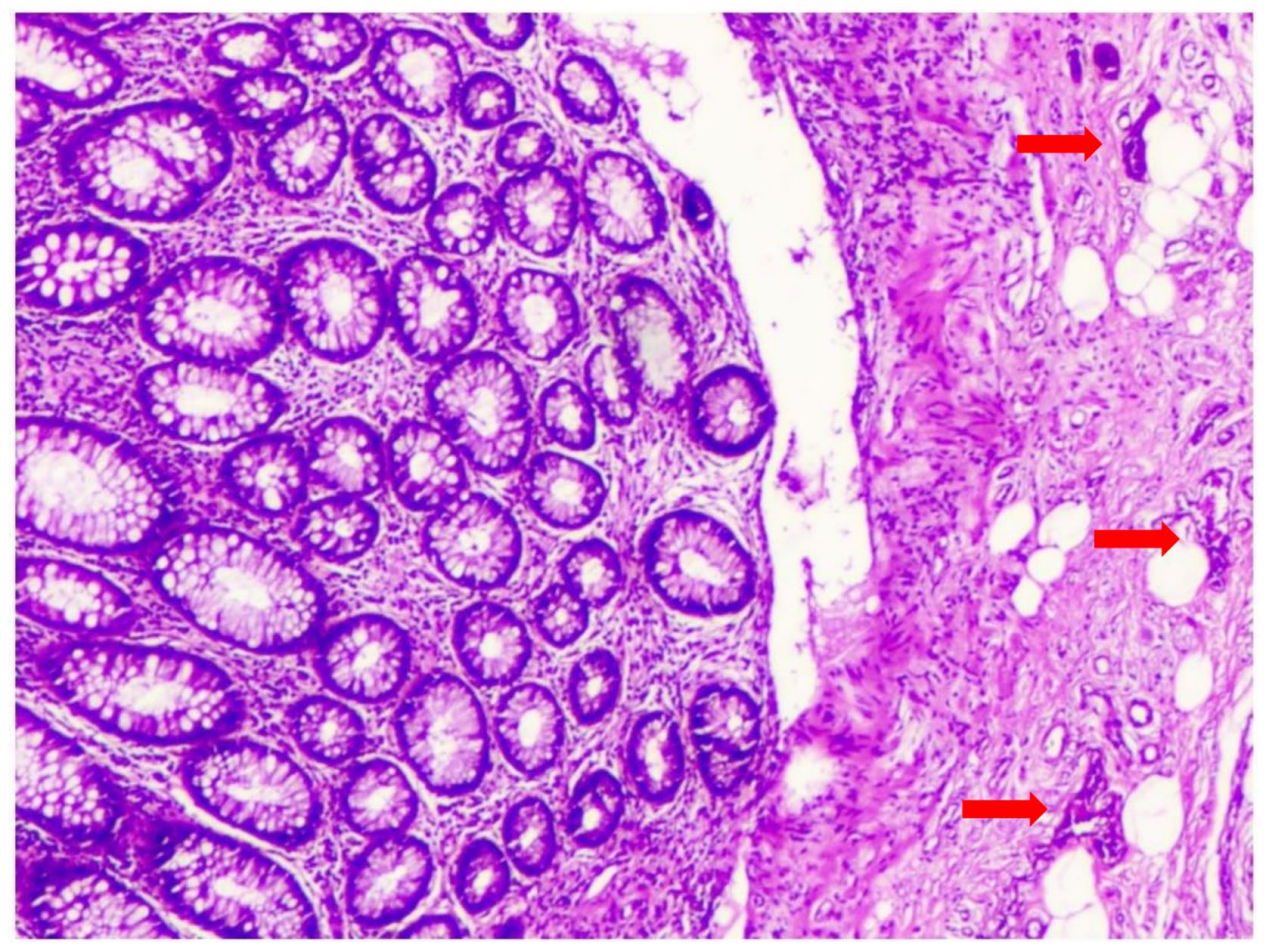
Postoperative histopathological analysis shows that GCC had infiltrated the muscularis propria.

Histopathological examination showed that GCC had invaded the muscularis propria (Figure 5), and all surgical borders were clear of any tumour; an immunohistochemical study suported the diagnosis by indicating positive results for Syn(+), CgA(+), CK20(+), CDX-2(+), Ki-67(about 15%), PMS2(+), MSH2(+), MSH6(+), MLHI(+), Villin(+), CK7(−), and PAS(+).

The tumor was classified as pT2N0M0 (stage IIA) in accordance with the American Joint Committee on Cancer (AJCC) 8th edition, and although the National Comprehensive Cancer Network (NCCN) guidelines suggested a right hemicolectomy, due to the patient’s advanced age, substantial comorbidities, and family’s preference, a combined decision was made to not proceed with more aggressive surgery. Instead, a planned and intensive surveillance plan was implemented, and at the 1-year follow-up, the patient remained in a clinically good condition, had normal tumor markers, and there was no radiological evidence of recurrence found in CT imaging.

## Discussion

The current case demonstrates a remarkable combination of three clinically rare occurrences: a femoral hernia that became incarcerated, an De Garengeot hernia, and an accidental finding of a GCC. This exceedingly uncommon coexistence makes it more clinically significant. Anatomically, femoral hernias pass through a narrower passage compared to inguinal hernias, making them much more likely to get incarcerated and strangulated, as demonstrated in our case. In addition, GCC is extremely rare in any context and is even more exceptional when it is found in a herniated appendix.

This uncommon occurrence also highlights a long-standing diagnostic difficulty. Pre-operative imaging, such as CT, can reliably detect the hernia and may even indicate appendiceal involvement; however, they are generally unable to tell the difference between benign inflammation and a hidden malignancy, as indicated in our case; consequently, thediagnosis has, to date, depended largely on postoperative histopathological examination. From a pathogenic point of view, one may speculate whether chronic mechanical irritation and repeated inflammation inside the restricted hernia sac may lead to mucosal hyperplasia and later malignant conversion. Although this idea is still a hypothesis, it implies that surgeons should maintain a heightened sense of suspicion for cancerous growths when they come across a firm or abnormally thick appendix in the situation of a recurring or trapped De Garengeot hernia.

GCC is a unique kind of tumor that has combined traits of adenocarcinoma, marked by goblet cell formation and mucin generation-and neuroendocrine transformation, and shows signs such as Syn and CgA (5). Based on its unique tissue structure, the growth pattern of GCC is considered to have biological behavior between standard appendicular adenocarcinoma and traditional carcinoid tumor. In clinical practice, diagnosis and classification depended solely on post operation histopathology, and in this patient, an immunohistochemical analysis verified both a gastrointestinal makeup (CK20+, CDX-2+) and neuroendocrine transformation (Syn+, CgA+). Existing grading methods divide GCC into three levels with higher grades—characterized by reduced tube-like structure—being associated with greater aggressiveness and an increased risk of lymph node and far-reaching metastasis. A histopathological analysis demonstrated invasion into the muscularis propria corresponding to pT2 stage. Notably, even pT1 cancer had a reported lymph node metastasis rate of 15–20% (6). Although several prognostic factors were observed in this patient, including negative margins, absence of lymph vessel or perineural spread, and complete mismatch repair protein expression, the observed Ki-67 index of approximately 15% suggested significant growth activity, and all of these factors presented a genuine clinical puzzle: a medium risk tumor having both good indicators and actual metastatic ability, making decisions about further treatment especially difficult.

From a surgical perspective, a single-stage operation that combined LA and TAPP repair was carried out successfully, and the success of this method in dealing with De Garengeot hernia relies largely on what is found during the operation and the existing classifications (7); therefore, meticulous examination of the appendix is essential, with careful assessment for swelling, tissue death, hardening, or growth, along with evaluation of the hernia sac for signs of infection. Drawing on the treatment strategy for Amyand’s hernia, based on the Losanoff and Basson classification, a one-stage repair using a synthetic mesh along with removing the appendix is usually acceptable for type I (normal appendix) and type II (acute appendicitis inside the hernia) cases (8). In our patient, the trapped appendix tip had no perforation or indications of peritonitis, which suggested an early, localized process; therefore, performing LA after completing the TAPP repair was believed to be both sensible and feasible, in line with the minimally invasive concept and preventing the need for a second surgery.

The main dispute arose after the operation, and for those with GCC at or above stage pT2, radical right hemicolectomy has always been the standard suggestion since it aims to thoroughly remove lymph nodes for precise staging and possibly a cure (9, 10). Nevertheless, in clinical practice, balancing the guideline suggestions with the individual situations of patients is necessary. However, the case represented a common yet challenging scenario, that is, an elderly patient with serious underlying comorbidities and a clear family opposition to further major surgery. This decision should not be regarded as ignoring the guidelines but rather as carefully adjusting the treatment objectives, shifting the emphasis from seeking theoretically curative thoroughness to attaining practical and personalized benefits for the particular patient in question.

When selecting such a non-standard management method, a strict and initiative follow up regime is of utmost importance; therefore, we implemented a well-designed surveillance program that involved periodic contrast-enhanced CT scans and tumor marker assessments. The absence of recurrence after 1 year is an encouraging early sign, providing initial support to this cautious strategy. Nevertheless, more robust evidence would be required to direct cases similar to this in future studies. Therefore, future studies should center on (1) creating prognostic models for gallbladder cancer (GCC) that include T stage, Ki-67 index, and lymphovascular invasion to better identify high risk patients; (2) probing into the function of new tools such as circulating tumor DNA (ctDNA) monitoring for earlier detection of recurrence; and (3) systematically gathering and sharing management experiences from similar cases to make possible significant meta-analyses and produce higher level of evidence for clinical decision making.

## Conclusion

For elderly patients having comorbidities and being in the early stage of GCC, a conservative approach focused on active surveillance might be a rational option after a meticulous discussion of risks and benefits, particularly when standard surgical suggestions are not possible. In the future, attention should be paid to improving GCC specific prognostic models, assessing new monitoring devices, and setting up cooperative registries to bolster the evidence for dealing with this clinically complex disease.

## Data Availability

The original contributions presented in the study are included in the article/supplementary material, further inquiries can be directed to the corresponding author.
